# The rate of adaptive evolution in animal mitochondria

**DOI:** 10.1111/mec.13475

**Published:** 2015-12-17

**Authors:** Jennifer E. James, Gwenael Piganeau, Adam Eyre‐Walker

**Affiliations:** ^1^ School of Life SciencesUniversity of SussexBrightonBN1 9QGUK; ^2^ UPMC Univ Paris 06UMR 7232Observatoire OceanologiqueAvenue de FontauléBP 44, 66651 Banyuls‐sur‐MerFrance; ^3^ CNRSUMR 7232Observatoire OceanologiqueAvenue de FontauléBP 44, 66651 Banyuls‐sur‐MerFrance

**Keywords:** adaptive evolution, distribution of fitness effects, genetic diversity, mitochondria, mutation rate, site frequency spectrum

## Abstract

We have investigated whether there is adaptive evolution in mitochondrial DNA, using an extensive data set containing over 500 animal species from a wide range of taxonomic groups. We apply a variety of McDonald–Kreitman style methods to the data. We find that the evolution of mitochondrial DNA is dominated by slightly deleterious mutations, a finding which is supported by a number of previous studies. However, when we control for the presence of deleterious mutations using a new method, we find that mitochondria undergo a significant amount of adaptive evolution, with an estimated 26% (95% confidence intervals: 5.7–45%) of nonsynonymous substitutions fixed by adaptive evolution. We further find some weak evidence that the rate of adaptive evolution is correlated to synonymous diversity. We interpret this as evidence that at least some adaptive evolution is limited by the supply of mutations.

## Introduction

Mitochondrial DNA (mtDNA) is widely used in evolutionary and ecological studies for a number of reasons. Firstly, a large number of copies of mtDNA are present in every cell, and this abundance made it relatively easy to use prior to the advent of PCR. Secondly, in many animal lineages mtDNA is highly variable due to its high mutation rate (for example, see Brown *et al*. 1979; Denver *et al*. 2000; Lynch 2010), allowing the analysis of evolutionary events over short timescales. Finally, mtDNA is inherited asexually, usually solely from the mother (Birky 1995), which means that it can be considered as a single locus, with all sites sharing a common genealogy. This can make some inferences easier to make because a single tree is appropriate for representing the evolutionary history of the molecule. This is particularly important in phylogeography, a field in which mtDNA is widely used to trace the geographical origins and movements of groups of individuals within species (for review of use in humans, see (Torroni *et al*. 2006). Because the use of mtDNA is so ubiquitous, the factors that govern its evolution have received considerable attention. In particular, the role of natural selection on the diversity and divergence of mtDNA has been a focus of research (Ballard & Whitlock 2004).

Initial studies that applied McDonald–Kreitman (MK) tests to mtDNA found an excess of nonsynonymous polymorphisms, suggesting that slightly deleterious mutations are common in mtDNA (Nachman 1998; Rand & Kann 1998). Slightly deleterious mutations contribute to polymorphism, but over time purifying selection is expected to remove them from populations, and so they are not expected to contribute substantially to between‐species variation in mtDNA. That purifying selection could be an important force on the evolution of mtDNA is intuitive, as mtDNA contains important genes, whose protein products are vital for the mitochondrial oxidative phosphorylation process (Ballard & Whitlock 2004). Multiple lines of evidence support the purifying selection hypothesis. For example, mitochondrial‐encoded genes experience base composition constraints due to the hydrophobic nature of mitochondrial‐encoded proteins (Naylor *et al*. 1995), mitochondrial proteins are highly conserved among mammal species (da Fonseca *et al*. 2008) and experimental work suggests that nonsynonymous changes in mtDNA can be eliminated very rapidly between generations (Fan *et al*. 2008; Stewart *et al*. 2008a,b).

There is also evidence to suggest that mtDNA might undergo adaptive evolution. In a study by Bazin *et al*. (2006), the authors failed to find the positive relationship between mitochondrial genetic diversity and effective population size expected under neutrality. They reasoned this could be due to widespread adaptive evolution on mtDNA. Using an MK test, the authors found evidence for adaptive evolution in mtDNA but not nuclear DNA in both vertebrates and invertebrates, although the signal was only significant for invertebrates. However, the reasons for the discrepancy between this study and previous results [e.g. Nachman (1998) and Rand & Kann (1998)] are unclear. Bazin *et al*. (2006) used a considerably larger data set than any previous analysis, which may have allowed them to identify the pattern of adaptive evolution. However, there are also some statistical concerns associated with this study. Bazin *et al*. (2006) used the Neutrality Index (NI) in order to quantify the strength and direction of selection from MK tests (Rand & Kann 1996) As a ratio of ratios, the neutrality index will tend to be biased and to have high variance (Stoletzki & Eyre‐Walker 2011). Also, studies that use the Neutrality Index must exclude data sets for which the index is undefined, which can produce an estimate of adaptive evolution which is biased upwards (Stoletzki & Eyre‐Walker 2011).

However, at least in particular systems there is good evidence to suggest that adaptive selection is an important influence on mitochondrial evolution.

Mitochondrial genes have a key role in the oxidative phosphorylation pathway, which provides a large proportion of a cell's required ATP. A change in metabolic demand may produce selection pressure on mitochondrial‐encoded genes to meet the metabolic demands of the host organism's lifestyle, possibly in concert with nuclear genes (Ballard & Rand 2005; Dowling *et al*. 2007). For example, evidence suggests that in bats, key genes in the oxidative phosphorylation pathway underwent adaptive evolution, likely as a consequence of the increased metabolic demand associated with the evolution of flight (Shen *et al*. 2010). There is also some evidence for a reduction in selective constraints on mtDNA in flightless bird species (Shen *et al*. 2009), which is in accordance with this hypothesis. There has also been adaptive evolution of mitochondrial genes in snakes, resulting in extensive modification of a number of core proteins, changes that may underlie the extreme metabolic regulation and efficiency exhibited by snakes (Castoe *et al*. 2008). Similarly, there is evidence of adaptive mitochondrial evolution in the simian (‘higher’) primates, which may be linked to the major phenotypic differences between simians and prosimians, which include a relatively large neocortex and a long lifespan (Doan *et al*. 2004; Grossman *et al*. 2004). Finally, da Fonseca *et al*. (2008) found evidence for functionally significant changes in mitochondrial‐encoded proteins across a range of mammal species.

In addition to experiencing direct selection, mitochondria may also undergo selective sweeps through hitchhiking with other maternally inherited genetic elements: these could include the sex chromosomes, if the female is the heterogametic sex, and cytoplasmically inherited symbionts (Ballard & Whitlock 2004). There is some evidence for reduced mtDNA diversity in birds (where females are heterogametic) compared with mammals, which could indicate mitochondrial hitchhiking with sex chromosomes (Berlin *et al*. 2007); however, the observed pattern could also be explained by other biological factors, such as differential mitochondrial mutation rates (Hickey 2008). Evidence for the effects of infection by cytoplasmically inherited symbionts on mtDNA is far less controversial, particularly for *Wolbachia*, a common symbiont of arthropods. *Wolbachia* infection is known to drive sweeps in mtDNA, as the infection is usually initially associated with only one mitochondrial haplotype as it spreads through a population. This has the effect of reducing mitochondrial diversity in the population and driving increased mitochondrial divergence between populations [for reviews, see (Hurst & Jiggins 2005; Galtier *et al*. 2009)].

In our analysis, we reconsider the question of whether mtDNA undergoes adaptive evolution. We address statistical concerns associated with removing undefined values from the data set using methods that are always defined for any informative data set. We also control for the presence of slightly deleterious mutations using two contrasting methods. In the first, we follow the nonparametric approach suggested by Messer & Petrov (2013), based on the insight of Fay *et al*. (2001), who suggested removing polymorphisms below a particular frequency when estimating the rate of adaptive evolution using MK‐type approaches in order to remove deleterious mutations segregating in the population. Although this is a useful ad hoc method, the cut‐off point for excluding polymorphisms is essentially arbitrary and theoretical work suggests that this method can still produce estimates of adaptive evolution that are biased downwards (Charlesworth & Eyre‐Walker 2008). Messer & Petrov (2013) suggested an extension of this method, in which the rate of adaptive evolution is estimated for each frequency class of polymorphism in the site frequency spectrum (SFS). As higher frequency variants are considered, the estimate of adaptive evolution should increase as more slightly deleterious mutations are excluded. At high polymorphism frequency classes, the rate of adaptive evolution is expected to reach an asymptote that is close to the true level of adaptive evolution. This method cannot be applied directly to mtDNA, because clonality makes the SFS highly erratic, and furthermore any one data set typically has too few polymorphisms to make inferences reliable. We therefore propose a method by which we can combine the divergence and SFS from different species in an unweighted and unbiased manner. We then investigate how our estimate of the rate of evolution changes as a function of the frequency of polymorphisms on this combined data set. We also apply a variant of the method suggested by Eyre‐Walker & Keightley (2009) and Boyko *et al*. (2008) to estimate the rate of adaptive evolution. Their methods use the SFSs at selected and neutral sites to infer the distribution of fitness effects (DFE) at the selected sites and then use this to make inferences about the level of adaptive evolution. Here, we estimate the DFE from the ratio of the SFSs at selected and neutral sites, combining data across species, and then use this to make inferences about rates of adaptive evolution.

## Methods

We used the data set originally compiled by Bazin *et al*. (2006), which was built using the Polymorphix database (Bazin *et al*. 2005), in order to conduct our analysis. For every species entry in the data set, we had two or more sequences from the ingroup species and between one and five outgroup species. We calculated levels of nonsynonymous and synonymous divergence using the method of Goldman & Yang (1994), as implemented in the codeml package of paml version 4.7 (Yang 2007) from pairwise alignments between each outgroup and a randomly chosen sequence from the ingroup. We also estimated the SFS and calculated polymorphism summary statistics for every species in the data set using our own scripts.

### Combining data

Our methods for estimating the rate of adaptive evolution required us to combine data across species. We did this by dividing the SFS at nonsynonymous and synonymous sites by the total number of polymorphisms, and the numbers of nonsynonymous and synonymous substitutions by the total number of substitutions to yield normalized SFSs and divergences estimates for each species and its outgroup; we then combined data across species. Hence if ${\hat{P}}_{\mathrm{ni}}\left(x\right)\;\text{and}\;{\hat{P}}_{\mathrm{si}}\left(x\right)$ are the observed numbers of nonsynonymous and synonymous polymorphisms at frequency *x* in the *i*th species the normalized values are

(1) $$\begin{array}{cc} {\hat{P}}_{\mathrm{ni}}^{*}\left(x\right)=\frac{{\hat{P}}_{\mathrm{ni}}\left(x\right)}{\sum_{\mathrm{all}\;x}{\hat{P}}_{\mathrm{ni}}\left(x\right)+\sum_{\mathrm{all}\;x}{\hat{P}}_{\mathrm{si}}\left(x\right)}\; & \\ {\hat{P}}_{\mathrm{si}}^{*}\left(x\right)=\frac{{\hat{P}}_{\mathrm{si}}\left(x\right)}{\sum_{\mathrm{all}\;x}{\hat{P}}_{\mathrm{ni}}\left(x\right)+\sum_{\mathrm{all}\;x}{\hat{P}}_{\mathrm{si}}\left(x\right)} \end{array}$$

and if ${\hat{D}}_{\mathrm{ni}}$ and ${\hat{D}}_{\mathrm{si}}$ are the numbers of nonsynonymous and synonymous substitutions between the *i*th species and its outgroup then the normalized values are

(2) $${\hat{D}}_{\mathrm{ni}}^{*}=\frac{{\hat{D}}_{\mathrm{ni}}}{{\hat{D}}_{\mathrm{ni}}+{\hat{D}}_{\mathrm{si}}}\;{\hat{D}}_{\mathrm{si}}^{*}=\frac{{\hat{D}}_{\mathrm{si}}}{{\hat{D}}_{\mathrm{ni}}+{\hat{D}}_{\mathrm{si}}}$$

The overall SFS and divergences were then calculated as

(3) $${\hat{P}}_{n}^{*}\left(x\right)=\sum_{\mathrm{all}\;i}{\hat{P}}_{\mathrm{ni}}^{*}\left(x\right)\;{\hat{P}}_{s}^{*}\left(x\right)=\sum_{\mathrm{all}\;i}{\hat{P}}_{\mathrm{si}}^{*}\left(x\right)$$

and

(4) $${\hat{D}}_{n}^{*}=\sum_{\mathrm{all}\;i}{\hat{D}}_{\mathrm{ni}}^{*}\;{\hat{D}}_{s}^{*}=\sum_{\mathrm{all}\;i}{\hat{D}}_{\mathrm{si}}^{*}$$

In this way we combine data across species weighting each species equally.

We estimate the rate of adaptive evolution using two statistics. The proportion of the nonsynonymous substitutions that are adaptive:

(5) $$\alpha =1-\frac{D_{s}P_{n}}{D_{n}P_{s}}$$

and the rate of adaptive evolution relative to the rate of mutation

(6) $$\omega_{a}=\frac{D_{n}}{D_{s}}-\frac{P_{n}}{P_{s}}$$

### Theoretical analysis

If we assume that the mutation rate is low (i.e. 2*N*_e_ *u* ≪ 1) and synonymous mutations are neutral, then the expected number of synonymous polymorphisms segregating in *i* of *n* sequences in a haploid is

(7) $$P_{s}\left(i\right)=\frac{2\theta }{i}$$

where θ* = N*_e_ *u*,* u* the rate of mutation per generation per site, and, in the case of mtDNA, *N*_e_ is the effective population size of females.

If we assume that all nonsynonymous mutations are deleterious (some can be sufficiently weakly selected to be neutral) and drawn from some distribution *G(S,**V***), where *S = *2*N*_e_ *s* and *s* is the strength of selection acting against a deleterious mutation, and ***V*** is a vector of parameters of the distribution then the expected number of nonsynonymous polymorphisms segregating in *i* of *n* sequences is

(8) $$P_{n}\left(i\right)=\theta \int_{0}^{\infty }\int_{0}^{1}G(S;V)H(S,x)Q(n,i,x)dxdS$$

where

$$\begin{array}{cc} H(S,x) & =2\frac{1-e^{S(1-x)}}{x\left(1-x\right)\left(1-e^{S}\right)}\\ & \;\text{and}\;\\ & Q\left(n,i,x\right)=\frac{n!}{i!(n-i)!}x^{i}{\left(1-x\right)}^{n-i} \end{array}$$

*H*(*S,x*) is the time a deleterious mutation subject to selection *S* spends at a frequency *x* and *Q*(*n,i,x*) is the probability of observing a mutation in *i* of *n* sequences for a mutation at frequency *x*. To fold the SFS we add *P*_n_(*i*) to *P*_n_(*n* − *i*), and *P*_s_(*i*) to *P*_s_(*n* − *i*). The ratio of the SFSs depends simply on the parameters governing the distribution of fitness effects.

The expected divergence at synonymous and nonsynonymous sites due to neutral and deleterious mutations is:

(9) $$D_{s}=2ut$$

(10) $$D_{n}=2ut\int_{0}^{\infty }G\left(S;V\right)\frac{-S}{1-e^{S}}dS$$

respectively, where −*S/*(1 − e^S^) is approximately *N*_e_ times the probability that a deleterious mutation will become fixed. The ratio *D*_n_/*D*_s_ depends solely on the parameters that govern the distribution of fitness effects.

We assume here that the distribution of fitness effects is a gamma distribution:

(11) $$G\left(S;\lambda ,\phi \right)=\frac{\phi^{\lambda }S^{\lambda -1}e^{-\phi S}}{\Gamma (\lambda )}$$

where λ is the shape parameter and ϕ the scale parameter of the gamma distribution; the mean of the distribution is λ/ϕ.

We use the equations above for two purposes. First we use the equations to study the consequences of folding the SFS on our ability to infer the rate of adaptive evolution. If the true value of α is α_true_ then to a good approximation the expected number of nonsynonymous substitutions, $D_{n}^{\prime }$, can be written as

(12) $$D_{n}^{\prime }=\frac{D_{n}}{1-\alpha_{\mathrm{true}}}$$

if we assume advantageous mutations are rare and strongly selected (i.e. they can contribute to divergence, but contribute little to polymorphism). The value of α we estimate using the polymorphisms from each frequency category is

(13) $$\alpha_{\mathrm{est}}\left(i\right)=1-\frac{D_{s}P_{n}\left(i\right)}{D_{n}^{\prime }P_{s}\left(i\right)}$$

### Estimating the rate of adaptive evolution

We can also use the equations above to estimate the rate of adaptive evolution. The ratio of the nonsynonymous and synonymous SFS is expected to depend solely on the parameters of the DFE under our model. We find the parameters of the DFE that minimize the squared difference between the observed and expected values of *P*_n_(*i*)/*P*_s_(*i*) using the Nelder–Mead algorithm as implemented in the Mathematica routine Minimize (i.e. we found the best fitting parameters using least squares). We take into account that in our data there are on average 3.52 as many nonsynonymous as synonymous sites. Using the inferred DFE we estimate the expected value of *d*_n_ */d*_s_ = ω_exp_ due to deleterious mutations, where *d*_n_ and *d*_s_ are the numbers of nonsynonymous and synonymous substitutions per site. If the observed value of *d*_n_ */d*_s_ = ω_obs_ is greater than this we infer that there has been adaptive evolution. We can estimate the rate of adaptive evolution relative to the rate mutation as

(14) $$\omega_{a}=\omega_{\mathrm{obs}}-\omega_{\exp}$$

and the proportion of substitutions that are adaptive as

(15) $$\alpha =\frac{\omega_{\mathrm{obs}}-\omega_{\exp}}{\omega_{\mathrm{obs}}}$$

### Independence

In our final analysis we consider the correlation between ω_a_ and synonymous diversity (π_S_), variables that are both calculated using the number of synonymous polymorphisms. As a consequence, ω_a_ and π_S_ are nonindependent and we would expect the variables to be positively correlated due to sampling error alone (see eqn (6)). To remove this statistical nonindependence, we split the synonymous polymorphisms into two independent groups by sampling from the hypergeometric distribution – in effect we randomly divide the synonymous sites into two equally sized groups. One group of synonymous polymorphisms is then used to estimate ω_a_, while the other is used to estimate π_S_ (This is equivalent to dividing our sequences into odd and even codons, as in; Smith & Eyre‐Walker 2003). This method removes the statistical nonindependence due to sampling error, and has the advantage that effective population size will be the same for both groups of synonymous polymorphisms: this is important as we are interested in determining the effect of *N*_e_ on rates of adaptive evolution.

## Results

To investigate rates of adaptive evolution in animal mitochondrial DNA we applied a variety of McDonald‐Kreitman type analyses to mtDNA data from a broad variety of animal species. We used the data set originally compiled by Bazin *et al*. (2006). This data set comprises multiple sequences from over 1000 animal species with at least one outgroup for each species. We excluded any species entry without at least one outgroup for which the synonymous divergence between the ingroup and outgroup was <0.1 or >1.0. We avoided outgroups that were very closely related to the ingroup because of problems in estimating the rate of adaptive evolution using MK‐type methods when divergences are short (Keightley & Eyre‐Walker 2012). We also avoided very divergent outgroups because of mutation saturation at nonsynonymous and synonymous sites, which leads to a tendency to underestimate the rate of nonsynonymous relative to the rate of synonymous substitution (Yang 2006; Dos Reis & Yang 2013). If multiple outgroups were available after this filtering we chose the most closely related outgroup. This left us with a data set of 514 animal species.

**Figure 1:**
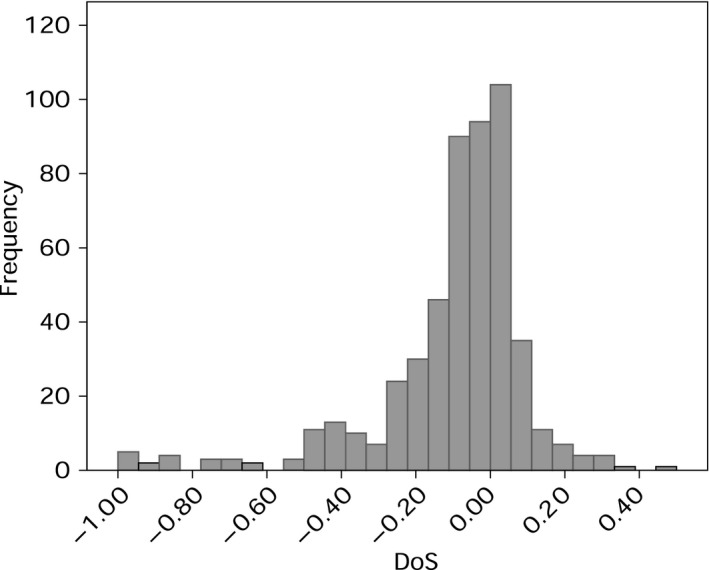
Histogram showing the frequency distribution of DoS values for the data set.

The Direction of Selection (DoS) statistic is a simple unbiased summary of the data in an MK table (DoS = *D*_n_/(*D*_n_ + *D*_s_) − *P*_n_/(*P*_n_ + *P*_s_)) (Stoletzki & Eyre‐Walker 2011) (note that throughout this study, *D*_x_ or *P*_x_ refer to the total number of substitutions and polymorphisms, at sites of type *x,* respectively, whereas *d*_x_ and *p*_x_ refer to the numbers per site). Assuming that synonymous mutations are neutral, positive values of DoS indicate a pattern of adaptive evolution at nonsynonymous sites, whereas negative values indicate the presence of slightly deleterious mutations. We find that DoS is negative for 347 of the 514 (68%) of species, indicating that in our data set slightly deleterious mutations are dominating mitochondrial evolution (Fig. 1). Qualitatively similar patterns are found in both vertebrates and invertebrates, and amongst the two largest invertebrate groups the arthropods and molluscs (Table 1). The median DoS value does not differ significantly between either vertebrates and invertebrates (Mann–Whitney *U*‐test *P* = 0.07), or between molluscs and arthropods (*P* = 0.632).

**Table 1:** A summary of the DoS results. The median DoS values and the proportion of DoS values that were negative (column titled ‘Prop negative DoS’) are given for each data set

Data set	*n*	Prop negative DoS	Median DoS
Complete dataset	514	0.68	−0.052
Vertebrates	404	0.70	−0.059
Invertebrates	110	0.59	−0.027
Arthopods	67	0.63	−0.030
Molluscs	25	0.60	−0.019

*n* = number of species included in each data set.

Although the majority of DoS values are negative, a considerable minority are positive. Of these positive values, only one, *Anolis punctatus*, is significantly positive after correcting for multiple tests (results given in Table 2); we estimate that ~80% of amino acid substitutions in this species have been fixed as a consequence of positive selection, but this is likely to be an overestimate due to the winner's curse (i.e. if evolution was rerun, we would expect to see a regression to the mean and a reduction in this high estimate).

**Table 2:** The species for which DoS is significantly positive, after correcting for multiple tests. The DoS value, the number of nonsynonymous and synonymous substitutions, the number of nonsynonymous and synonymous polymorphisms, and an estimate of α for the species are given, followed by the *P*‐value calculated using Fisher's exact test

Species	DoS Value	*D*_n_	*D*_s_	*P*_n_	*P*_s_	α	Fisher's Exact Test (*P*‐value)
*Anolis punctatus*	0.36	21.47	14.63	29	97	0.80	>0.001

The above analyses indicate that slightly deleterious mutations dominate the evolutionary dynamics of mtDNA, and slightly deleterious mutations can obscure adaptive evolution in MK‐type analyses. Therefore we sought to estimate the rate of adaptive evolution while controlling for the presence of deleterious mutations.

Deleterious mutations are expected to segregate in the population at low frequencies. Therefore, we investigated how the estimate of adaptive evolution changes as a function of increasing frequency category of polymorphisms. We expect the estimate of adaptive evolution to increase and to eventually approach an asymptote as the frequency category of polymorphism being considered increases, because with increasing frequency category, more segregating deleterious mutations are removed from the population (Fay *et al*. 2001; Charlesworth & Eyre‐Walker 2006; Messer & Petrov 2013). Messer & Petrov (2013) suggest estimating the rate of adaptive evolution from the asymptote. However, it should be noted that they advocate using the unfolded SFS, which were not able to do because of difficulties in inferring ancestral states in most of our species. Unfortunately, there are challenges in applying this method in the mtDNA of individual species for two reasons. Firstly, most species in the data set have insufficient numbers of polymorphisms; and secondly, as mtDNA is largely clonal, all mtDNA sites share the same genealogy and hence the site frequency spectra tend to be highly erratic. Therefore we devised a method to combine data across multiple species.

The method requires that all data sets have the same number of sampled chromosomes and so we first randomly subsampled the number of chromosomes for each species down to 12 chromosomes, excluding species without sufficient numbers of sequenced chromosomes. We chose to reduce the data to 12 chromosomes because theoretical analyses suggest that this is the minimum sample size that is likely to yield a reasonable asymptote (results not shown). This subsampling reduced our total data set to 372 species. We then combined the folded SFS and divergence data across all species from the reduced data set, in a manner that weights the data for all species equally (see Methods). Finally we calculated our measure of adaptive evolution for each frequency category of polymorphism using the combined SFSs and divergence data. Following Messer & Petrov (2013) we fit a curve of the form *y* = *a* + *b* e^−cx^ to the data by least squares, where *a*,* b* and *c* are parameters that are estimated; the estimate of adaptive evolution was taken as the value from this curve for the highest frequency class – i.e. by setting *x* in the equation above to 6. Confidence intervals were obtained by bootstrapping the data by species. We performed the estimation for both α, the proportion of nonsynonymous substitutions inferred to have been fixed by positive adaptive evolution, and ω_a_, the rate of adaptive nonsynonymous substitution relative to the rate of mutation. The two statistics gave qualitatively similar patterns.

**Figure 2:**
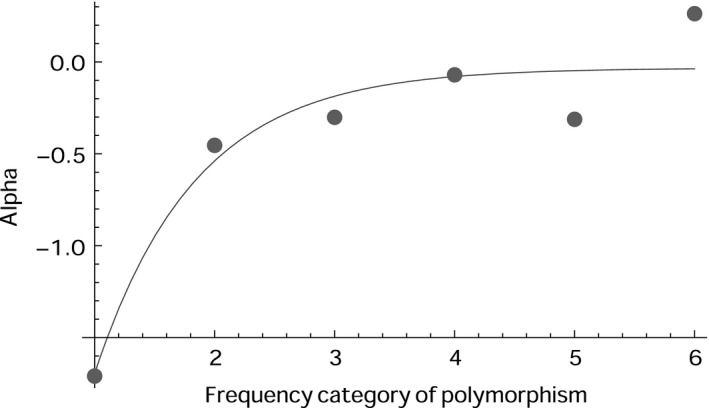
Graph of our estimate of α (using the Messer–Petrov method) plotted against the frequency category of polymorphism. An asymptotic curve of the form *y* = *a* + *b* *e* ^−cx^ was fitted to the data in order to obtain an estimate of α.

As expected, we find that our estimate of α is negative for low frequency classes and that it increases as the frequency of polymorphisms increases. However, our graph appears to asymptote at a value close to 0 when all the data are considered together, suggesting that there is little evidence of adaptive evolution in mtDNA (Fig. 2, Table 3). The confidence interval on this estimate is surprisingly large given the number of data sets we have analysed. If we divide the data set up into vertebrates and invertebrates, as Bazin *et al*. (2006) did, we find that the estimate of α is positive for invertebrates, and for the two biggest groups of invertebrates, arthropods and molluscs; however none of these estimates are significantly greater than zero, and there are no significant differences between any of the groups.

**Table 3:** Results table showing estimates of α calculated using a variant of the Messer–Petrov method. α was estimated by fitting an exponential function to the graph of α plotted against polymorphism frequency category. A bootstrap was performed 100 times in order to calculate the 95% confidence intervals (CIs). The ‘Prop < 0’ column gives the proportion of bootstrap data sets in which the estimate of α was less than zero, providing one‐tailed *P*‐values for our results. Results are shown for the complete data set, a ‘low divergence data set’ that included species for which 0.1 < d_s_ < 0.5, and for vertebrates, invertebrates, arthropods and molluscs separately

Dataset	α	Lower 95% CI	Upper 95% CI	Prop < 0
Complete data set	−0.037	−0.4	0.28	0.58
Low divergence dataset	−0.21	−1.13	0.62	0.68
Vertebrates	−0.24	−0.62	0.21	0.83
Invertebrates	0.26	−0.16	0.57	0.08
Arthopods	0.23	−0.32	0.56	0.09
Molluscs	0.44	−6.36	0.87	0.30

There may be two reasons we may have underestimated the rate of adaptive evolution using the asymptotic method. First, some of our species pairs are quite divergent and d_n_/d_s_ tends to be lower in highly divergent species (Dos Reis & Yang 2013). To investigate this possibility, we repeated our analysis on a data set that was restricted to those species pairs for which 0.1 < d_s_ < 0.5. Unfortunately this reduced our data set to just 83 species. In this reduced data set we again found no evidence of adaptive evolution (Table 3)**.** Therefore it does not appear that the lower value of α is due to the underestimation of d_n_/d_s_ on long branches.

Second, the low values of α could be due to the fact we have used the folded SFS. We folded the SFS because most of our outgroup species are too divergent to allow us to infer ancestral states. Unfortunately, folding the SFS is expected to yield a greater underestimation of α than not folding the SFS (Charlesworth & Eyre‐Walker 2008). The potential severity of the underestimation can be estimated using the theory set out in Charlesworth & Eyre‐Walker (2008) (see Methods). In the model we assume that synonymous mutations are neutral and that nonsynonymous mutations are either deleterious or strongly beneficial. The fitness effects of deleterious nonsynonymous polymorphisms are drawn from a gamma distribution and can be sufficiently weakly selected that they are effectively neutral. We parameterize the model such that it yields the observed value of the number of nonsynonymous polymorphisms per site relative to the number of synonymous polymorphisms per site from the combined 12 chromosome data set – i.e. we selected a shape parameter for the gamma distribution, and find the mean strength of selection acting on the deleterious mutations that will yield the correct value of *p*_n_ */p*_s_.

In Table 4 we give the estimate of α obtained from the last frequency class (this is a good approximation to the asymptotic value obtained by fitting a curve of the type used above), when 12 chromosomes have been sampled for various DFEs that are consistent with the data. It is evident from this analysis that estimates of α using the folded SFS are much more downwardly biased than estimates using the unfolded SFS and that the underestimate can be very substantial, particularly if there is little adaptive evolution and the DFE is platykurtic (i.e. high shape parameter values); for example, the estimate of α from the whole data set (−0.037) is consistent with a true α value of 20% if the DFE has a shape parameter of 0.5 and true α value of 0.39 if the DFE has a shape parameter of 0.75.

**Table 4:** The predicted estimated values of α (α_est_) using the Messer–Petrov method under different distributions of fitness effects that are consistent with the data (Shape = shape parameter of the DFE, Mean *S* = mean strength of selection), calculated for three different true values of α

Shape	Mean *S*	α_True_	α_est_ (folded)	α_est_ (unfolded)
0.25	810 000	0	−0.18	−0.037
0.5	3600	0	−0.39	−0.071
0.75	770	0	−0.64	−0.011
0.25	810 000	0.25	0.11	0.22
0.5	3600	0.25	−0.043	0.20
0.75	770	0.25	−0.23	0.17
0.25	810 000	0.5	0.41	0.48
0.5	3600	0.5	0.30	0.46
0.75	770	0.5	0.18	0.45

The tendency for the asymptotic method to underestimate the true value of α when the SFS is folded motivated the development of an alternative method. Several parametric methods have already been developed to estimate rates of adaptive evolution in which the distribution of fitness effects is estimated from the SFS, and this is then used to make inferences about the rate of adaptive evolution (Boyko *et al*. 2008; Eyre‐Walker & Keightley 2009). Unfortunately, none of these methods can be applied to our data because of the way in which we have constructed our unweighted average SFSs and divergences. We therefore developed a new method in which we estimated the DFE from the ratio of the SFS at selected sites and the SFS at neutral sites (i.e. the *p*_n_/*p*_s_ values for each frequency category) using least squares. We then used the DFE to estimate the expected value of d_n_/d_s_ due to neutral and slightly deleterious mutations, inferring adaptive evolution if the observed value of d_n_/d_s_ exceeded the observed value. The value of α and ω_a_ can easily be estimated from considering the difference between the observed and expected values of d_n_/d_s_. This method is similar to the second method presented by Eyre‐Walker & Keightley (2009) which uses the method of Eyre‐Walker *et al*. (2006) to estimate the DFE. This and the current method assume that any demographic or sampling process affects the nonsynonymous and synonymous sites similarly – e.g. a demographic process that reduces synonymous singletons by 20% also reduces nonsynonymous singletons by the same amount. In reality the demography does affect the ratio of the selected and neutral SFSs and hence the estimate of the DFE (Otto & Whitlock 1997), but simulations suggest the method is fairly robust (Eyre‐Walker *et al*. 2006).

**Figure 3:**
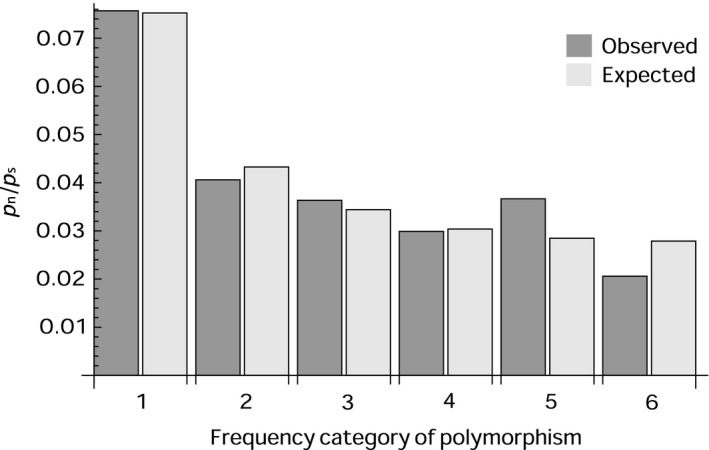
Bar chart of observed and expected values of *p*_n_/*p*_s_, as predicted from fitting a distribution to the DFE to the complete data set, plotted against the frequency category of polymorphism.

Using this new parametric method substantially increases our estimates of α over those obtained by the asymptotic method, and many are now significantly greater than zero (Table 5). For the entire data set we estimate that 26% of all nonsynonymous substitutions have been fixed by positive selection (*P* < 0.001). The estimate of α is higher in invertebrates, (α = 0.45, *P* < 0.001) than vertebrates (α = 0.14, *P* = 0.16) but the difference is not significant. Interestingly, DFEs estimated from most of the data sets are remarkably similar. These DFEs fit the observed values of *p*_n_ */p*_s_ well – the fit of the DFE to the whole data set is shown in Fig. 3.

**Table 5:** Results table showing estimates of α calculated using the parametric method

Dataset	α	Lower 95% CI	Upper 95% CI	Prop < 0	Shape	Mean *S*
All	0.26	0.057	0.45	0	0.44	4600
Low divergence dataset	0.058	−0.91	0.57	0.49	0.27	140 000
Vertebrates	0.14	−0.18	0.42	0.16	0.45	3200
Invertebrates	0.45	0.12	0.61	0	0.44	7500
Arthropods	0.41	0.23	0.65	0	0.44	6900
Molluscs	0.61	−1.6	0.93	0.22	0.39	15 000

The data was bootstrapped 100 times to calculate 95% CIs; the proportion of bootstrap data sets in which the estimate of α was less than zero is given in the ‘Prop < 0’ column. The shape parameter of the DFE is given in the ‘Shape’ column, while the mean strength of selection is given in the ‘Mean *S*’ column.

Results are shown for the complete data set, a ‘low divergence data set’ that included species for which 0.1< d_s_ < 0.5, and for vertebrates, invertebrates, arthropods and molluscs separately.

We expect species with larger *N*_e_ to undergo more adaptive evolution than species with small *N*_e_ if the rate of adaptive evolution is limited by the supply of mutations. Such a correlation has been described for nuclear DNA across a small number of species (Gossmann *et al*. 2012). Unfortunately, we do not have estimates of *N*_e_ for most of our species as we do not have an estimate of the mutation rate per generation. However, previous analyses have suggested that synonymous diversity (π_S_) in mtDNA is correlated to *N*_e_ (Piganeau & Eyre‐Walker 2009). To investigate whether that was the case in this data set we split *P*_s_ into two independent variables by sampling from a hypergeometric distribution. *P*_s1_ was used to estimate π_S_ in each species and *P*_s2_ was used to estimate a measure of the effectiveness of selection, *P*_n_ */(P*_n_ + *P*_s_). We find that *P*_n_/*(P*_n_ + *P*_s_) is significantly and negatively correlated to π_s_ (Spearman's ρ = −0.47, *P* < 0.0001), suggesting that synonymous diversity is correlated to *N*_e_.

To investigate whether the rate of adaptive evolution is correlated to *N*_e_, we used the data from the above analysis, in which *P*_s_ was split into two halves: one half was used to estimate π_s_ and the other to estimate the rate of adaptive evolution using our parametric method. Up to this point in our analysis we have primarily concentrated on α, the proportion of substitutions that are advantageous, because it has a readily understood interpretation. However, differences in α between species can be due to differences in either the number of advantageous substitutions or the number of effectively neutral substitutions; this is not ideal for this analysis since both might be expected to be correlated to *N*_e_. We therefore estimated ω_a_, the rate of adaptive nonsynonymous substitution relative to the rate of mutation (Gossmann *et al*. 2012). In order to use our method of calculating ω_a_, it was necessary to divide the species used in the analysis into groups. We divided our species evenly into groups based on the synonymous diversity of each species: ω_a_ and average π_S_ could then be calculated for each group. We find that the correlation between ω_a_ and π_S_ is always positive, however, the statistical significance of the correlation changes depending on the number of groups used; we only find a significant Spearman's correlation when we use 4 and 8 groups (Table 6). This is likely to be because the data is noisy. We conclude that there is some weak evidence that rates of adaptive evolution are correlated to levels of synonymous diversity in mitochondria.

**Table 6:** The strength and statistical significance of the correlation between ω_α_ and π_S_. Results for both Spearman's and Pearson's tests are shown. In order to calculate ω_α_ using our method, it was necessary to group the species used in this analysis: the number of groups is given in the first column of the table

Number of groups	Spearman's coefficient	*P*‐value	Pearson's coefficient	*P*‐value
4	1	0.042	0.94	0.065
6	0.54	0.27	0.4	0.43
8	0.86	0.007	0.48	0.23
10	0.26	0.47	0.52	0.13
12	0.45	0.15	0.53	0.077

## Discussion

We have investigated the evolutionary dynamics of mitochondrial evolution using variations of the McDonald‐Kreitman test, in which the number of substitutions (i.e. differences between species) are contrasted to the number of polymorphisms (i.e. differences within species) at two categories of sites; those at which most mutations are neutral and those at which selection acts. Using this approach we find, as others have (Ballard & Kreitman 1994; Nachman 1998; Rand & Kann 1998; Nabholz *et al*. 2008) that the evolution of mtDNA is dominated by slightly deleterious mutations. However, when we control for these slightly deleterious mutations by estimating the distribution of fitness effects, we find evidence that mitochondria generally experience non‐negligible level of adaptive evolution, with 26% of nonsynonymous substitutions fixed by positive selection. In this regard, our results broadly agree with the findings of Bazin *et al*. (2006) who found evidence of adaptive evolution in animal mitochondria, particularly in invertebrates, using the neutrality index.

We also find some weak evidence that the level of adaptive evolution in mitochondria is correlated to the effective population size, as measured by the level of synonymous genetic diversity. This is expected if the rate of adaptation is limited by the supply of mutations because species with a high *N*_e_ are more likely to generate the advantageous mutations that allow them to adapt. It should be noted here that we expect a correlation between ω_a_ and *N*_e_ but not necessarily between ω_a_ and π_s_, because π_s_ is expected to be equal to *N*_e_ *u*, while ω_a_ is expected to be independent of the mutation rate. Hence variation in the mutation rate per generation will generate noise in the correlation between ω_a_ and π_s_. The observed correlation between ω_a_ and π_s_ is not expected under a model in which all adaptation comes from standing genetic variation; under this model species with high and low diversity must adapt at the same rate. If not, then the less diverse species must be waiting for advantageous mutations to occur and hence be limited in their adaptation by the supply of mutations. The correlation between the rate of adaptive evolution and the level of neutral diversity is consistent with the results of Gossmann *et al*. (2012) who observed a correlation between the rate of adaptive evolution and the effective population size in the nuclear genes of 13 independent pairs of animal, fungal and plant species.

Bazin *et al*. (2006) have suggested that there is little variation in the *N*_e_ of mtDNA between animal species because groups of animals with apparently very different census population sizes have similar synonymous diversities. However, Popadin *et al*. (2007) and Piganeau & Eyre‐Walker (2009) have found evidence of variation in the effective population size of mtDNA between species, by showing that there is significant correlation between a measure of the effectiveness of selection and a correlate of *N*_e_, a result we have confirmed by showing that *P*_n_/(*P*_n _+ *P*_s_) is significantly negatively correlated to synonymous diversity after accounting for the nonindependence between these variables.

Our major result, that mitochondria undergo substantial levels of adaptive evolution, appears to be inconsistent with previous work indicating that nuclear genes in regions of low recombination undergo little or no adaptive evolution, at least in *Drosophila* (Betancourt *et al*. 2009; Campos *et al*. 2014). This is thought to be due to Hill‐Robertson interference (HRi), whereby selection at one site interferes with selection at linked sites. There might be several reasons why mitochondria undergo adaptive evolution while nonrecombining nuclear loci do not. First, although we have estimated that a substantial proportion of nonsynonymous substitutions have been due to adaptive evolution in mtDNA, the absolute rate is low; the average value of d_n_/d_S_, estimated from the sum(normalized d_n_)/sum(normalized d_s_) = 0.036 so our overall estimate for ω_α_ = 0.009. This is considerably lower, for example, than the estimate in the nuclear genes of *Drosophila melanogaster* and *yakuba* [ω_a_ = 0.050 estimated from 6120 autosomal genes (D. Castellano, unpublished results)], and is perfectly consistent with the relationship between ω_a_ and the rate of recombination that is observed in *Drosophila* (Castellano *et al*. 2015). Second, animal mitochondria typically have small genomes (Boore 1999) which will limit the total rate of deleterious and advantageous selection, and hence the level of HRi.

Third, mitochondria might show relatively high levels of adaptive evolution because the genes they contain are essential for cell survival, and so we might predict mutations to be under intense selection, which will reduce the effects of HRi. The reason is as follows. The more strongly selected an advantageous mutation is, the more likely it is to escape HRi even if the deleterious mutations are also more strongly selected, because, assuming there is no epistasis, the mutation load exerted by deleterious mutations is independent of the fitness effect of deleterious mutations (Haldane 1937). Mitochondria are also inherited in an unusual manner, experiencing what has been termed the mitochondrial bottleneck during germ cell development (Stewart & Larsson 2014), which could act to expose variants to selection. This possibility is supported by experimental work which demonstrates that purifying selection can have a rapid and drastic impact on mtDNA, removing deleterious mutations in very few generations (Fan *et al*. 2008; Stewart *et al*. 2008a,b).

It should be noted that our analysis assumes that synonymous mutations are effectively neutral. This may not be the case if mitochondria experience selection on synonymous codon usage for translational accuracy or efficiency. However, there is little evidence for this phenomenon in mitochondria (Pesole *et al*. 1999; Sun *et al*. 2009; Castellana *et al*. 2011). Our analysis is also limited in that we only consider individual protein coding sequences for each species as opposed to complete mitochondrial genomes, which were not available for the majority of species used in this analysis. We were also not able to take into account possible differences between the rates of adaptive evolution of different mitochondrial proteins, for which there is some evidence (Meiklejohn *et al*. 2007; da Fonseca et al. 2008). However, our approach should be robust to demographic effects and to sampling error associated with alignment size, as these factors are expected to affect both synonymous and nonsynonymous sites equally. Importantly, this study has a major advantage over previous work in that our estimates of α should be unbiased, whereas past methods gave estimates of α that were biased downwards, even if a correction was made to remove deleterious polymorphisms from the analysis.

In summary, we have found evidence that mtDNA undergoes substantial amounts of adaptive evolution and that the rate of adaptive evolution is correlated to the diversity of the species being considered. These results have important implications for molecular ecology. MtDNA is widely used as a neutral genetic marker; however, our results indicate that up to 45% of nonsynonymous substitutions could be fixed by positive selection, a figure that rises to over 60% if we restrict our results to invertebrates. Therefore, adaptive evolution is likely to have a nontrivial impact on mitochondrial diversity. MtDNA diversity will, at least in part, reflect the amount of time since the last selective sweep, rather than demographic processes affecting the population.

A.E.‐W. conceived the study, G.P. acquired and processed the sequence data, J.E.J. and A.E.‐W. performed the analysis and wrote the manuscript.

## Data accessibility

Divergence, SFS, DoS and α estimates, in addition to all alignment files used in this analysis, are available to download from Figshare at: http://dx.doi.org/10.6084/m9.figshare.1408490.
